# Causes of hospitalisation among a cohort of people with HIV from a London centre followed from 2011 to 2018

**DOI:** 10.1186/s12879-021-06082-y

**Published:** 2021-04-29

**Authors:** Sophia M. Rein, Fiona C. Lampe, Clinton Chaloner, Adam Stafford, Alison J. Rodger, Margaret A. Johnson, Jeffrey McDonnell, Fiona Burns, Sara Madge, Alec Miners, Lorraine Sherr, Simon Collins, Andrew Speakman, Andrew N. Phillips, Colette J. Smith

**Affiliations:** 1 Institute for Global Health, University College London (UCL), Royal Free Campus, Rowland Hill St, NW3 2PF, London, UK; 2 Royal Free London NHS Foundation Trust, London, UK; 3 HIV i-Base, London, UK; 4 London School of Hygiene and Tropical Medicine (LSHTM), London, UK

**Keywords:** HIV, AIDS, Hospitalization, Morbidity, Causes, Diagnoses

## Abstract

**Background:**

We describe the spectrum of ICD-10 classified causes for hospitalisations occurring between 2011 and 2018 in a cohort of people living with HIV (PLHIV).

**Methods:**

This sub-study includes 798 PLHIV participating in the Antiretroviral, Sexual Transmission Risk and Attitudes (ASTRA) questionnaire study who were recruited from a large London centre. A medical record review identified the occurrence and causes of hospitalisation from the date of questionnaire completion (February–December 2011) until 1 June 2018. Up to five causes were classified by an HIV clinician using the ICD-10 system.

**Results:**

There were 274 hospitalisations in 153 people (rate = 5.8/100 person-years; 95% CI: 5.1, 6.5). Causes were wide-ranging; the most common were circulatory (16.8%), digestive (13.1%), respiratory (11.7%), infectious diseases (11.0%), injury/poisoning (10.6%), genitourinary diseases (9.9%) and neoplasms (9.1%). A tenth (27/274) of hospitalisations were related to at least one AIDS-defining illness. Median duration of hospitalisation was 5 days (IQR 2–9). At the time of hospitalisation, median CD4 count was high (510 cells/μl; IQR: 315–739), while median CD4 nadir was relatively low (113 cells/μl; IQR: 40–239). At admission, half of individuals (51%) had a previous AIDS-defining illness and 21% had viral load > 50 copies/ml. Individuals admitted for infectious diseases were particularly likely to have unfavourable HIV-related clinical characteristics (low CD4, viral non-suppression, not on antiretroviral therapy (ART), previous AIDS).

**Conclusions:**

In the modern combination antiretroviral therapy era, the spectrum of causes of hospitalisation in PLHIV in the UK is wide-ranging, highlighting the importance of holistic care for PLHIV, including prevention, early detection and treatment of comorbidities.

## Background

Following the introduction of effective combination antiretroviral therapy (ART) in 1996, AIDS-related mortality and morbidity among people living with HIV (PLHIV) in high-income countries declined dramatically [1, 2]. As life expectancy of PLHIV has continued to increase [3, 4], chronic conditions related to aging are increasingly prevalent [5, 6]. This is reflected in the causes of hospitalisation among PLHIV, with non-AIDS conditions now accounting for an increasing proportion [7, 8].

Monitoring the causes of hospitalisation among PLHIV in detail gives insight into changing patterns of morbidity, emerging new causes and current and future healthcare needs. However, few recent studies have comprehensively evaluated the causes of hospitalisation in PLHIV in the contemporary treatment era, and, to our knowledge, none from the UK.

We investigated hospitalisations during a six-to-seven-year period among 798 PLHIV recruited from a London centre in 2011. We previously reported on predictors of all-cause hospitalisation in this cohort [9]. Based on a detailed individual record review, we now describe the spectrum of ICD-10 classified causes of hospitalisations, and patient and clinical characteristics at admission. This descriptive analysis can improve our understanding of the current pattern of morbidity among PLHIV, and inform health care planning and interventions.

## Methods

The Antiretrovirals, Sexual Transmission Risk and Attitudes (ASTRA) questionnaire study recruited PLHIV from eight HIV outpatient clinics in England from February 2011 to December 2012 [10] (North West London research ethics committee 10/H0720/70).

A sub-study of hospitalisations was undertaken for participants from the Royal Free Hospital, London. Of 3258 ASTRA participants, 899 (28%) were recruited at the Royal Free Hospital, of whom 809 (90%) consented to additional linkage of questionnaire data with routine clinical data [10] and 798 with follow-up data available were included. Data on hospitalisations occurring in this cohort from the date of questionnaire completion (February–December 2011; baseline) until 1 June 2018 was obtained using routine clinic data enhanced by a detailed review of electronic and paper Royal Free hospital medical records. Information was extracted on all admissions documented (including admissions at hospitals other than the Royal Free): dates of admission and discharge, the admitting hospital, whether the admission was classified as an emergency and the causes. In order to maximise data regarding causes of hospitalisation, data were extracted from a number of sources. This included patient discharge summaries, patient notes and clinic letters. Causes were then assigned an appropriate ICD-10 code by a clinician who reviewed all available information regarding an admission. Up to five causes were assigned to every hospitalisation, therefore numbers can sum to more than 100%. Hospitalisations were defined as overnight stays at the hospital. Repeated hospitalisations from individuals were included.

We previously reported on the association of baseline factors with subsequent all cause hospitalisation [9]; this report presents a descriptive analysis of the causes of hospitalisation and patient characteristics of those admitted for the most common causes.

## Results

Of the 798 individuals, 74% were men who have sex with men (MSM), 9.5% were heterosexual men and 16.3% were women. Median age (IQR) at baseline was 46 (40–51) years; median CD4 count (IQR) at baseline was 621 (441–820); 719 (92%) were on ART at baseline and 653 (82%) had a suppressed viral load (≤50 copies/ml).

Over a median follow-up of 6.4 years (IQR 6.0–6.6), 274 hospitalisations occurred in 153 out of 798 people (19%), corresponding to an overall rate of 5.8/100 person-years (95% CI: 5.1–6.5). There were a total of 17 deaths (2.1%) over the follow-up time. In total, 104 (13%) individuals were lost to follow-up, which we defined as those who had their last clinic visit more than 18 months before end of the study in June 2018 (last clinic visit up to December 2016) and did not die during the period between the last visit and end of follow-up. Overall, 206 (75%) hospitalisations were emergencies; 223 (81%) took place at the Royal Free Hospital. Of those hospitalised, 97 (63%) were hospitalised once, 31 (20%) twice, 6 (4%) three times and 19 (12%) four or more times. The rate of subsequent admissions among those previously hospitalised was 64.2/100 person-years (95% CI: 52.7–75.7). Median time to re-admission was 237 days (IQR 54–583); 17 (18%) re-admissions occurred within 30 days of discharge.

**Fig. 1:**
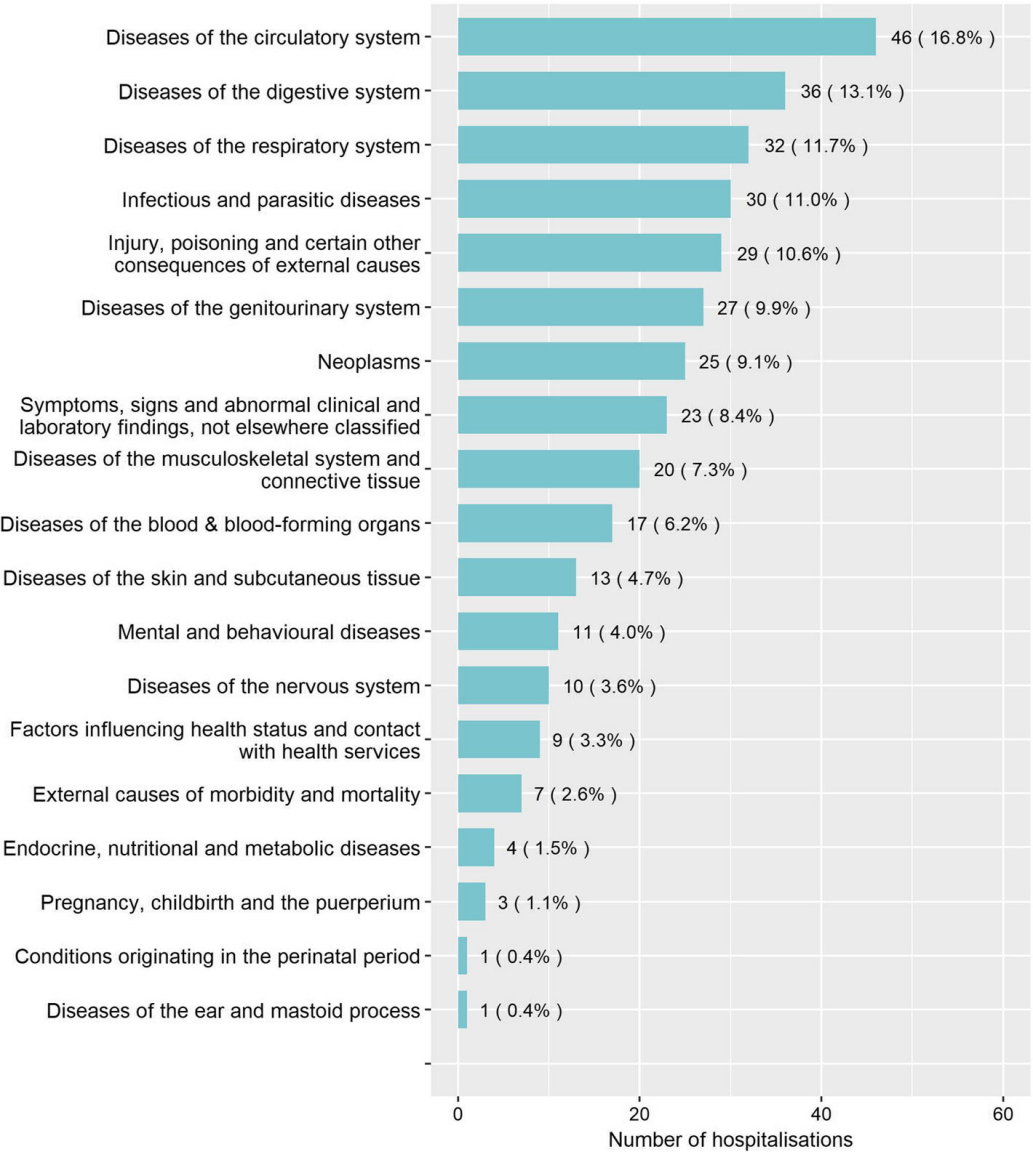
Number and percentage of hospitalisations related to each class of ICD-10 classified causes (denominator is the total number of hospitalisations (274)). Percentages sum to > 100% as multiple causes could be documented for a single hospitalisation

Of the 274 hospitalisations, 174 had one cause assigned, 69 had two, 14 had three, 9 had four, 5 had five causes and no information was available for 3 (415 causes in total). Figure 1 shows the distribution of causes. The seven most common ICD-10 categories, which cover at least one cause for 196 (72%) hospitalisations, are described below.

Circulatory diseases were the most commonly documented causes, involving 16.8% (*n* = 46) of admissions. This included: heart disease (*n* = 20 including: infective and acute pericarditis, mitral valve insufficiency, cardiomyopathy, cardiac arrest, cardiac arrhythmias, and heart failure); ischaemic heart disease (*n* = 10 including: angina pectoris, myocardial infarctions and chronic ischaemic heart disease); pulmonary embolism (*n* = 5); cerebrovascular disease (*n* = 3); atherosclerosis and other diseases of the arteries/arterioles (*n* = 4); diseases of veins, lymphatic vessels and lymph nodes (n = 4). Other causes (*n* = 2) were chronic rheumatic heart disease (mitral valve disease) and hypertension.

Digestive diseases were the second most documented cause, present in 13.1% (*n* = 36) of hospitalisations. Most common were diseases of: liver (*n* = 8 including: alcoholic hepatitis, hepatic failure, liver cirrhosis); gallbladder, biliary tract and pancreas (*n* = 6); intestines (*n* = 7 including: anal abscesses, fistula of intestines, constipation, megacolon); oesophagus, stomach and duodenum (*n* = 4); other digestive diseases (n = 7). There were three admissions due to non-infective enteritis and colitis, three due to acute peritonitis, two due to appendicitis, and one due to hernia.

Respiratory diseases were documented for 11.7% of admissions (*n* = 32). The specific conditions were: influenza and pneumonia (*n* = 20 including: seasonal flu, bacterial pneumonia, viral pneumonia); chronic lower respiratory diseases (*n* = 9 including: chronic obstructive pulmonary disease, asthma); unspecified acute lower respiratory infection (*n* = 5); other respiratory diseases (n = 5); upper respiratory infections (*n* = 3).

In 11.0% of hospitalisations (*n* = 30), infectious diseases were a documented cause. This included HIV-related infections (*n* = 10 including: mycobacterial infection, cytomegalovirus, *Pneumocystis jirovecii* pneumonia, tuberculosis, encephalopathy, wasting syndrome, haematological and immunological abnormalities); STIs (*n* = 6: syphilis, gonorrhoea, Chlamydia, anogenital warts); protozoal diseases (*n* = 5); intestinal infectious diseases (n = 5), mycoses (*n* = 4). Other causes (*n* = 11) were related to herpes zoster, infections of central nervous system, other bacterial diseases or infectious agents, TB of nervous system.

Of the hospitalisations for injury, poisoning and other consequences of external causes (*n* = 29, 10.6%), poisoning by drugs, medicines and biological substances was listed in 16 cases. Four were related to antiretrovirals; other substances included: non-steroidal anti-inflammatory drugs (NSAID), antiepileptic, sedative-hypnotic, antidepressants, psychostimulants, anticoagulants, calcium-channel blockers, antacids and anti-gastric secretion drugs, oxytocic drugs. Five of the hospitalisations due to poisoning were additionally recorded as intentional self-harm by self-poisoning with drugs/alcohol. Eight hospitalisations were related to complications of surgical and medical care including: infection or other procedure complications, and complications of internal orthopaedic prosthetic devices, implants and grafts. There were 19 injury-related causes (head, neck, thorax, abdomen, lower back, lumbar spine and pelvis, elbow and forearm, hip and thigh, knee and lower leg, ankle and foot and foreign body in the genitourinary tract).

For 27 (9.9%) admissions for genitourinary diseases, documented causes were: diseases of the urinary system (*n* = 7 including: urinary tract infections and urethral fistulas), renal tubule-interstitial diseases (*n* = 5); diseases of male genital organs (n = 5). Other causes included renal failure, urolithiasis, non-inflammatory disorders of female genital tract and glomerular disease.

There were 25 hospitalisations (9.1%) related to neoplasms; the majority (87% of causes) were malignant. The most common affected regions of those were: digestive organs (*n* = 10), ill-defined secondary and unspecified sites (*n* = 6), respiratory and intrathoracic organs (*n* = 4), genital organs (*n* = 2 female and *n* = 1 male), Kaposi’s sarcoma (n = 1), melanoma (n = 1), lip, oral cavity and pharynx (n = 1). Non-malignant neoplasms were documented for four admissions.

The remaining 12 ICD-10 categories were each mentioned in less than 9% of the total admissions (Fig. 1).

Of note, there were 11 hospitalisations related to mental health problems (additional to the 5 self-poisoning cases described above). Four were related to depressive episodes; three to use of alcohol, opioids or other stimulants; one to non-drug/alcohol induced delirium; four unspecified.

In total, 28 hospitalisations (10%) had one or more AIDS-defining illness (ADI) documented: pneumonia (*n* = 12); encephalopathy (*n* = 3); *Mycobacterium avium* complex (*n* = 2); lymphoma (n = 2); cryptococcal meningitis (n = 2); Kaposi sarcoma (n = 2); pneumocystis pneumonia (n = 2); cytomegalovirus (n = 1); tuberculosis and toxoplasmosis (n = 1); wasting syndrome (n=1).

Demographic, clinical and other key characteristics of patients at the time of their admission are presented in Table 1. At admission, median age was 52 years (IQR 46–60); median CD4 count was 510/μl (IQR 315–739); median CD4 nadir was 113/μl (IQR 40–239); median viral load: 738 copies/ml (IQR:162–22,653). Half (51%) of hospitalised individuals had a previous AIDS diagnosis and 21% had viral non-suppression at admission.

**Table 1:** Characteristics of individuals at time of hospitalisation, number of emergency admissions and duration of hospital stay according to most common causes

	ICD-10 classified cause of hospitalisation: 7 most common causes and total							
Characteristics	Circulatory diseases	Digestive diseases	Respiratory diseases	Infectious and parasitic diseases	Injury/poisoning /other external causes	Genitourinary diseases	Neoplasms	Total admissions
**N**	46	36	32	30	29	27	25	274
**Demographic group**^b^								
MSM	34 (74%)	27 (75%)	21 (66%)	19 (63%)	16 (55%)	13 (48%)	17 (68%)	175 (64%)
Black African heterosexual men	0 (0%)	2 (5.6%)	0 (0%)	4 (13%)	0 (0%)	4 (15%)	1 (4.0%)	14 (5.1%)
Other heterosexual men	7 (15%)	5 (14%)	5 (16%)	5 (17%)	5 (17%)	4 (15%)	3 (12%)	38 (14%)
Black African women	0 (0%)	1 (2.8%)	0 (0%)	1 (3.3%)	2 (6.9%)	2 (7.4%)	2 (8.0%)	14 (5.1%)
Other women	5 (11%)	1 (2.8%)	6 (19%)	1 (3.3%)	6 (21%)	4 (15%)	2 (8.0%)	33 (12%)
**Median age** (IQR)^a^	56 (46–64)	52 (46–58)	53 (47–67)	54 (45–60)	48 (43–59)	53 (46–60)	54 (48–60)	52 (46–60)
**Median CD4 count** in cells//μl (IQR)^a^	511 (343–804)	340 (272–620)	465 (347–753)	378 (170–660)	525 (449–696)	528 (356–893)	410 (267–608)	510 (315–739)
**Median CD4 count nadir** cells/μl (IQR)^a^	60 (39–250)	102 (44–133)	65 (12–185)	59 (18–138)	202 (50–280)	117 (26–147)	74 (14–153)	113 (40–239)
**Virally non-suppressed** (> 50 copies/ml)^a^	3 (7%)	5 (14%)	9 (28%)	14 (47%)	8 (28%)	3 (11%)	8 (32%)	57 (21%)
**Median years since HIV diagnosis** (IQR)^a^	20 (11–25)	17 (13–24)	17 (11–26)	15 (13–20)	12 (8–22)	17 (13–23)	21 (13–26)	18 (11–23)
**Currently not on ART**^a^	2 (4.4%)	0 (0%)	1 (3.1%)	3 (10%)	1 (3.5%)	1 (3.7%)	4 (16%)	13 (4.7%)
**Median years since first started ART** (IQR)^a^	15 (7–20)	15 (11–19)	14 (9–19)	13 (9–17)	8 (6–14)	15 (12–20)	16 (11–20)	14 (9–19)
**Prior AIDS diagnosis**^a^	31 (67%)	18 (50%)	18 (56%)	22 (73%)	8 (28%)	12 (44%)	14 (56%)	141 (51%)
**Most recent smoking status**^c^								
Current smoker	19 (41%)	10 (29%)	14 (44%)	13 (43%)	10 (36%)	6 (22%)	6 (24%)	110 (40%)
Ex-smoker	8 (17%)	12 (35%)	9 (28%)	8 (27%)	8 (29%)	6 (22%)	12 (48%)	69 (26%)
Never smoked	19 (41%)	12 (35%) missing = 2	9 (28%)	9 (30%)	10 (36%) missing = 1	15 (56%)	7 (28%)	92 (34%) missing = 3
**Emergency hospitalisations**	37 (80%)	30 (83%)	31 (97%)	26 (87%)	24 (83%)	20 (74%)	10 (40%)	206 (75%)
**Median duration of admission in days (IQR)**	6 (3–8)	6 (3.5–10.5)	6 (3–9)	7 (4–12)	4 (2–7)	4 (2–8)	8 (3–18)	5 (2–9)

*IQR* interquartile range; *ART* antiretroviral therapy; *MSM* men who have sex with men. ^a^Defined from clinical records; ^b^Defined from ASTRA questionnaire responses and classified according to the definition used for PLHIV by Public Health England [22]; ^c^Defined from clinical records where available, otherwise defined from ASTRA questionnaire

For injury/poisoning, more women (28%), particularly Black African women (21%), were hospitalised compared to the proportion overall (17 and 12% respectively). Those admitted for infectious and genitourinary diseases were more likely to be heterosexual men (30% in both categories), particularly of Black African ethnicity, compared to those admitted for other causes. Median age at hospitalisation was somewhat lower for injury/poisoning than the other common causes (Table 1).

Individuals hospitalised for digestive, infectious diseases or neoplasms had lower median CD4 counts compared to other causes. A prior AIDS diagnosis was much less prevalent in those hospitalised for injury/poisoning (28%) compared to other common causes and hospitalisations overall (51%); similarly, CD4 nadir was higher for the injury/poising cause. Almost half of individuals admitted for infectious diseases had detectable viral load at hospitalisation compared to 21% of individuals admitted overall. Individuals admitted for circulatory, respiratory or infectious diseases were more likely to be current smokers compared to other causes.

For ADI hospitalisations, the median (IQR) current and nadir CD4 counts at admission were 560 (339–700) and 69 (17–202) cells/μl respectively, 26 (96%) were on ART and 11 (41%) had viral load> 50 copies/ml. The median time since HIV diagnosis was 20 years (IQR:13–26).

The median duration of hospitalisation was 5 days (IQR 2–9), varying from 4 to 8 across the seven common causes (Table 1).

## Discussion

The causes of hospitalisation among a cohort of PLHIV followed up from 2011 to 2018 were wide-ranging with the most common being circulatory, digestive, respiratory, infectious diseases, injury/poisoning, genitourinary diseases and neoplasms in that order. AIDS-defining conditions accounted for 10% of hospitalisations.

Two other European studies of PLHIV in the contemporary era found infections were the most common cause of hospitalisation, with non-AIDS infections at least as common as AIDS-related. A French study in PLHIV in 2011 found that non-AIDS infections were the most common cause of hospitalisation (16.4%), followed by HIV-related diseases (15.6%) [11]. A Spanish study also found that infectious diseases (35%, of which 36% were AIDS-related) were the most common causes of hospitalisation in 2003–13, followed by digestive and respiratory diseases, hepatic decompensations, non-AIDS malignancies, psychiatric illnesses and cardiovascular diseases (CVD) [12]. A global systematic review among PLHIV focusing primarily on the period 2007–2015 found that the most common admission causes for adults were ADIs (31% in Europe) and bacterial infections (27% in Europe) in all geographic regions. Other common causes in Europe were respiratory (14%), psychiatric (13%), cardiovascular (12%), renal (11%), and liver diseases (10%) [7].

The spectrum of causes of hospitalisation in our study are similar to those found for mortality in PLHIV in recent studies in high income settings [13–15], including a study of 206 deaths of PLHIV in London in 2016, suggesting a similar underlying pattern of morbidity not dominated by AIDS-defining illnesses [13].

In comparison to the 2018/19 National Health Service England data on hospitalisation in the general population [16], infections and circulatory disease admissions accounted for a larger proportion in our study of PLHIV, while pregnancy and childbirth were less common, which is expected given the lower proportion of women. Digestive diseases, neoplasms, respiratory diseases, injury/poisoning, circulatory, genitourinary and musculoskeletal diseases, and symptoms, signs and abnormal clinical findings, were among the top ten causes both in our study and in the general population, with some difference in relative importance [16]. Although the comparison is complicated by demographic differences, this suggests the pattern of morbidity causing hospitalisation may be broadly similar in PLHIV as in the general population, with persisting differences in admissions for infectious diseases.

Half of individuals in our study had previous AIDS at hospitalisation; this percentage was particularly high for admissions with infectious diseases as a cause, but was also over 50% for circulatory, digestive, respiratory, and neoplasm causes. Overall, 43% of those hospitalised had previous AIDS at baseline (2011) compared to 34% in the whole study population. Our previous findings showed that lower baseline CD4 nadir predicted hospitalisation in this population [9]. The potential long-lasting effects of immunosuppression on a range of morbidities beyond ADIs specifically [17] reinforces the importance of timely diagnosis and treatment for HIV.

The rate of hospitalisation of 5.8/100 person-years in our study population was lower than some other recent studies in other high income settings [11, 18, 19]. Our study population had a high median CD4 count at baseline (621 (IQR: 441–820)) and included a low proportion of individuals with recent diagnosis (at baseline (2011) only 3.6% had been diagnosed within the past year) for whom hospitalisation rates are particularly high [20].

Although the main demographic groups of PLHIV in the UK (MSM, heterosexual men and women) are represented in our study population, compared to Public Health England (PHE) estimates of the diagnosed population of PLHIV in the UK in 2011 [21] (our study baseline), our population is comprised of a higher proportion of MSM (74% in our study vs 44% in UK), older individuals (aged > 50: ~ 30% vs 23%) and those with higher CD4 counts (CD4 ≥ 500: 68% vs. 51%). These differences may affect the relative frequency of hospitalisation causes observed in our study. For example, there may be some underrepresentation of immunodeficiency-related and overrepresentation of age-related conditions.

Hospitalisations occurring at hospitals other than the Royal Free may have been missed if not reported to the HIV physician by the patient, general practitioner or hospital, or if this information was not documented. ASTRA participants may differ from non-participants with respect to levels and patterns of morbidity (899 patients completed questionnaires of 1336 at the Royal Free invited to participate (response rate of 67%)).

## Conclusions

In summary, in the contemporary ART era, there is a wide spectrum of causes for hospitalisation in PLHIV in the UK not dominated by AIDS-related causes. These findings highlight the importance of holistic care, including the prevention, early detection and treatment of common chronic conditions.

## Data Availability

Any personally identifiable data cannot be made publicly available to protect participants’ privacy. All other relevant data are available upon request to the senior author (contact: f.lampe@ucl.ac.uk).

## Abbreviations

ADI: AIDS-defining illness
ART: Antiretroviral therapy
CI: Confidence interval
CVD: Cardiovascular disease
IQR: Interquartile range
MSM: Men who have sex with men
NSAID: Non-steroidal anti-inflammatory drugs
PLHIV: People living with HIV
